# Supplementary material for: Syncytial germline architecture is actively maintained by contraction of an internal actomyosin corset
Source: Nat Commun. 2018 Nov 8;9:4694. doi: 10.1038/s41467-018-07149-2 (PMC6224597; doi:10.1038/s41467-018-07149-2)
Supplement: Supplementary file 1 — Supplementary Information [file 41467_2018_7149_MOESM1_ESM.pdf]

Supplementary Information

Syncytial germline architecture is actively maintained by contraction of an internal actomyosin corset

Priti et al.

Supplementary Figures

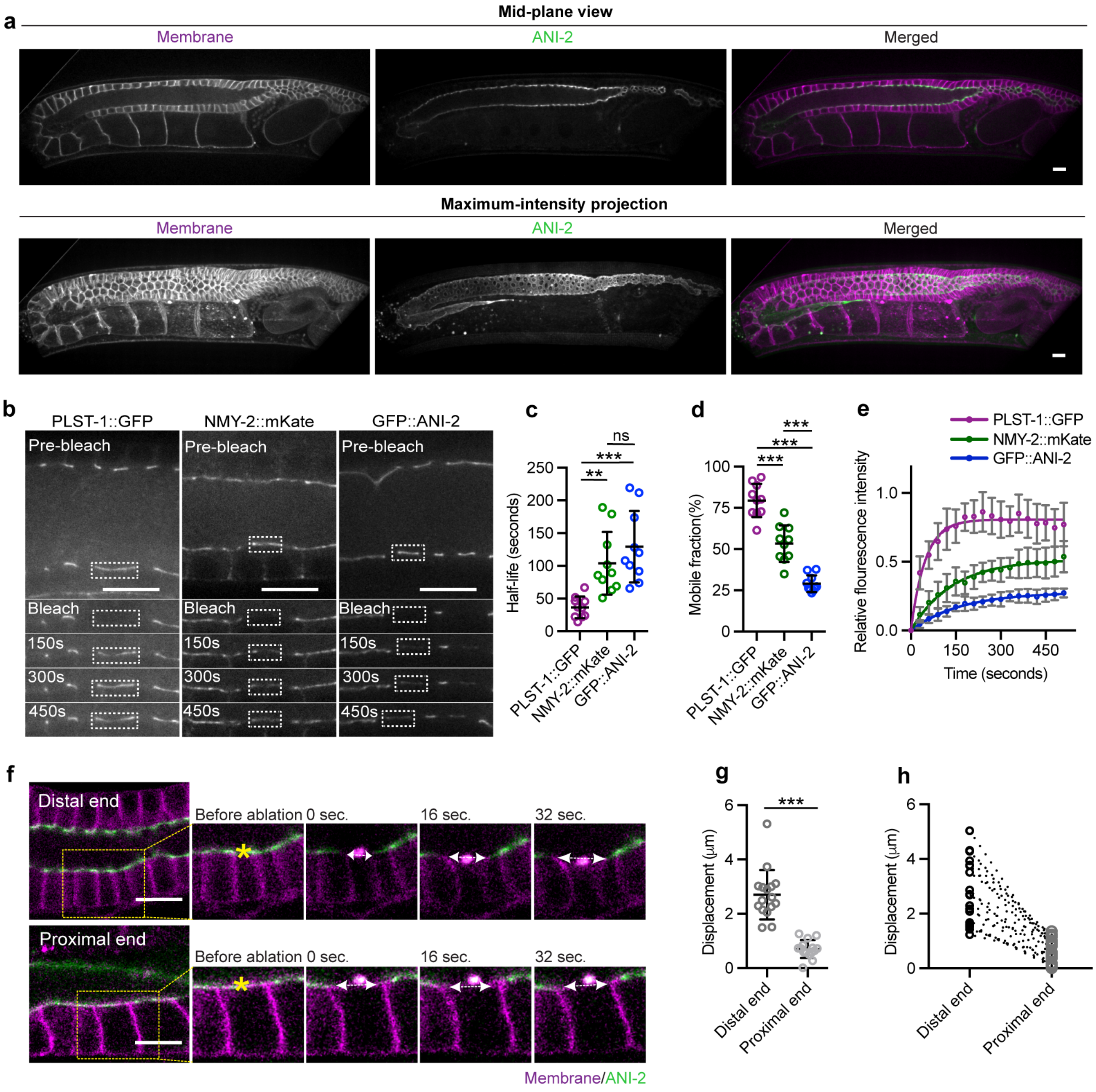

Supplementary Figure 1: Further characterization of the syncytial germline inner actomyosin corset. **(a)** Mid-plane and maximum-intensity projected views of the entire *C. elegans* germline expressing mCherry::PH;GFP::ANI-2. **(b)** Time-lapse images of PLST-1::GFP, NMY-2::mKate and GFP::ANI-2 analyzed before and after photobleaching (white rectangle box) **(c-e)** Quantification of the mobile fraction, half-life and recovery kinetics of PLST-1::GFP, NMY-2::mKate and GFP::ANI-2 ($N = 10$ for each). **(f)** Representative image of the point ablations made at the rachis bridge of the distal and proximal region of the gonad expressing mCherry::PH;GFP::ANI-2. Asterisk indicates the site of point ablation. White double-headed arrow shows the displacement at each time point after ablation. **(g, h)** Quantification of total displacement after laser ablation at the rachis bridge of different (distal, $N = 16$; proximal, $N = 15$) gonads and same gonad ($N = 19$). For the laser ablation in same gonad, the ablation was done in random order at the distal and proximal end. Data are represented as mean ± SD (***$p < 0.0001$, **$p < 0.005$, ns- non significant). Statistical analysis was done using one-way ANOVA test **(c-e)** and Mann Whitney U-test **(g)**, N represents number of gonads analyzed and scale bar indicates, 10 μm.

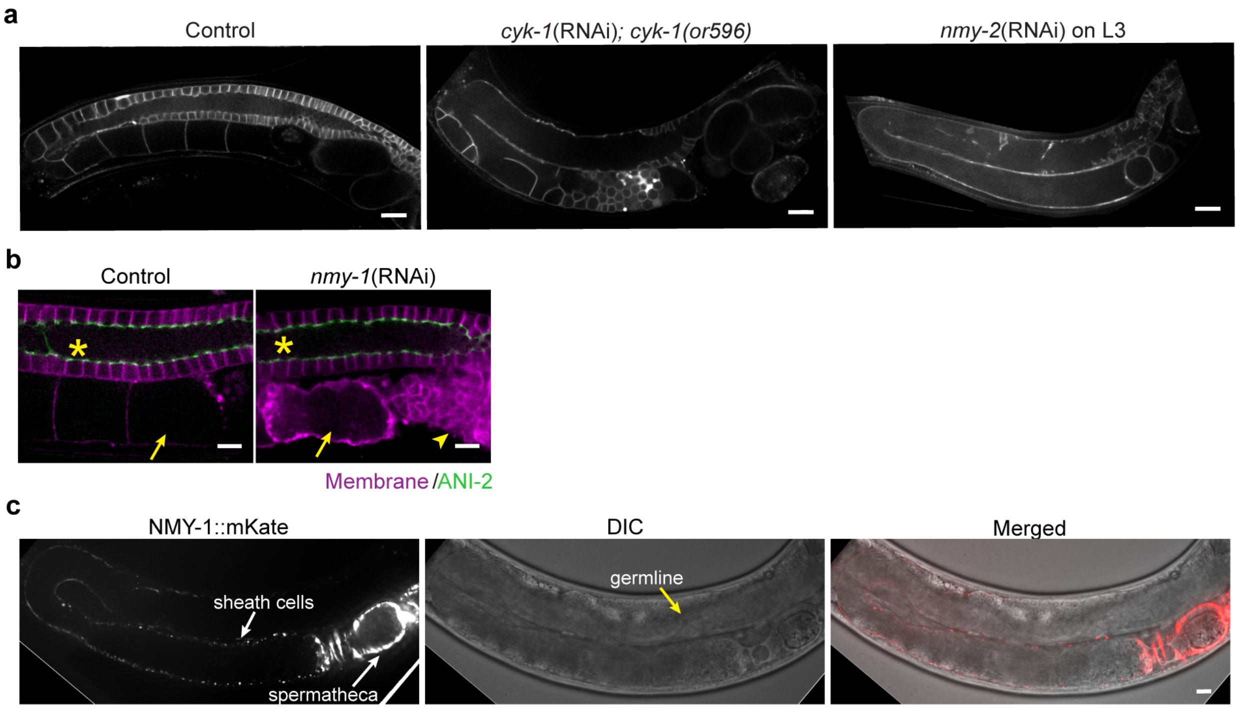

Supplementary Figure 2: Phenotypes of complete depletion of the formin CYK-1, myosin-II NMY-2, and myosin-II NMY-1. **(a)** Confocal image of the entire germline of control, *cyk-1(RNAi)* on *cyk-1(or596)* mutants at 20°C and *nmy-2(RNAi)* on younger worms, L3 stage, expressing a membrane marker (mCherry::PH) **(b)** Confocal image of germline expressing mCherry::PH;GFP::ANI-2 with control and *nmy-1(RNAi)*. Knockdown of NMY-1 did not show any defect in the syncytial germline architecture (yellow asterisk). However, unlike the proper oocytes in control, *nmy-1(RNAi)* worms had defective oocytes (yellow arrows) and embryos trapped in the spermatheca (yellow arrowhead) ($N = 20$). **(c)** Endogenous localization of CRISPR/Cas9 knock-in generated NMY-1::mKate. It shows strong expression at the sheath cells and spermatheca (white arrows) but is completely absent from the germline (yellow arrow in the DIC image). Scale bar indicates 10 μm.

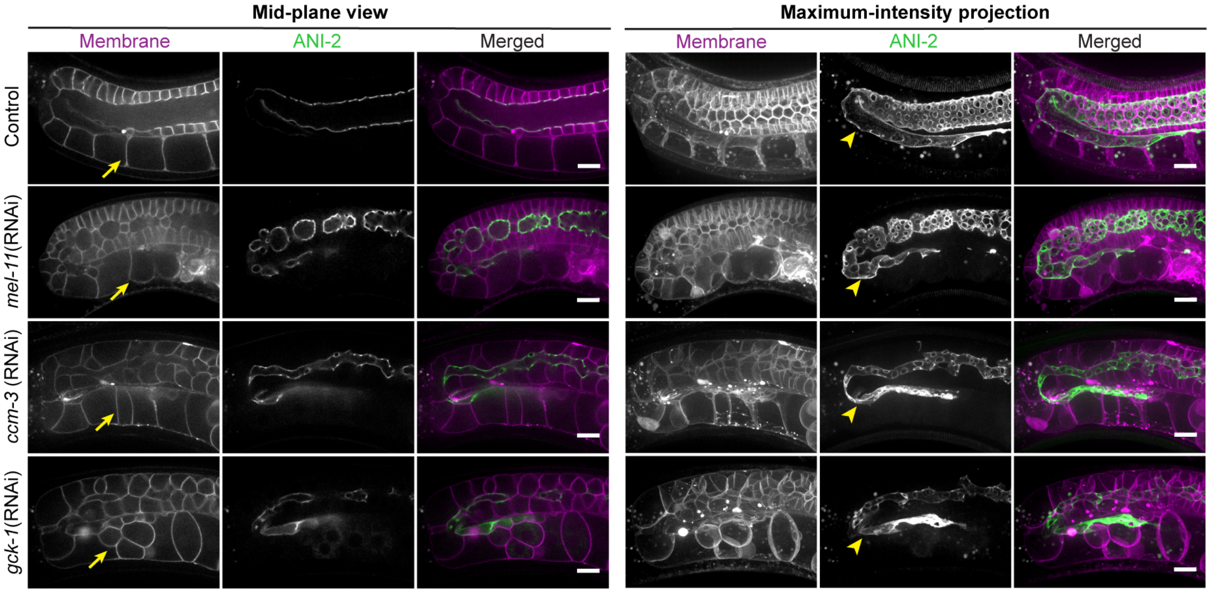

Supplementary Figure 3: Excessive contractility leads to formation of smaller oocytes. Mid-plane and maximum intensity projection views of the loop region of control, *mel-11(RNAi)*, *ccm-3(RNAi)* and *gck-1(RNAi)* gonads expressing GFP::ANI-2 (green) and membrane marker (magenta). Unlike the control, *mel-11(RNAi)*, *ccm-3(RNAi)* and *gck-1(RNAi)* gonads were sterile with small oocytes at the proximal end (yellow arrows) and hyper-constricted rachis (yellow arrowheads). Scale bar indicates 10 μm.

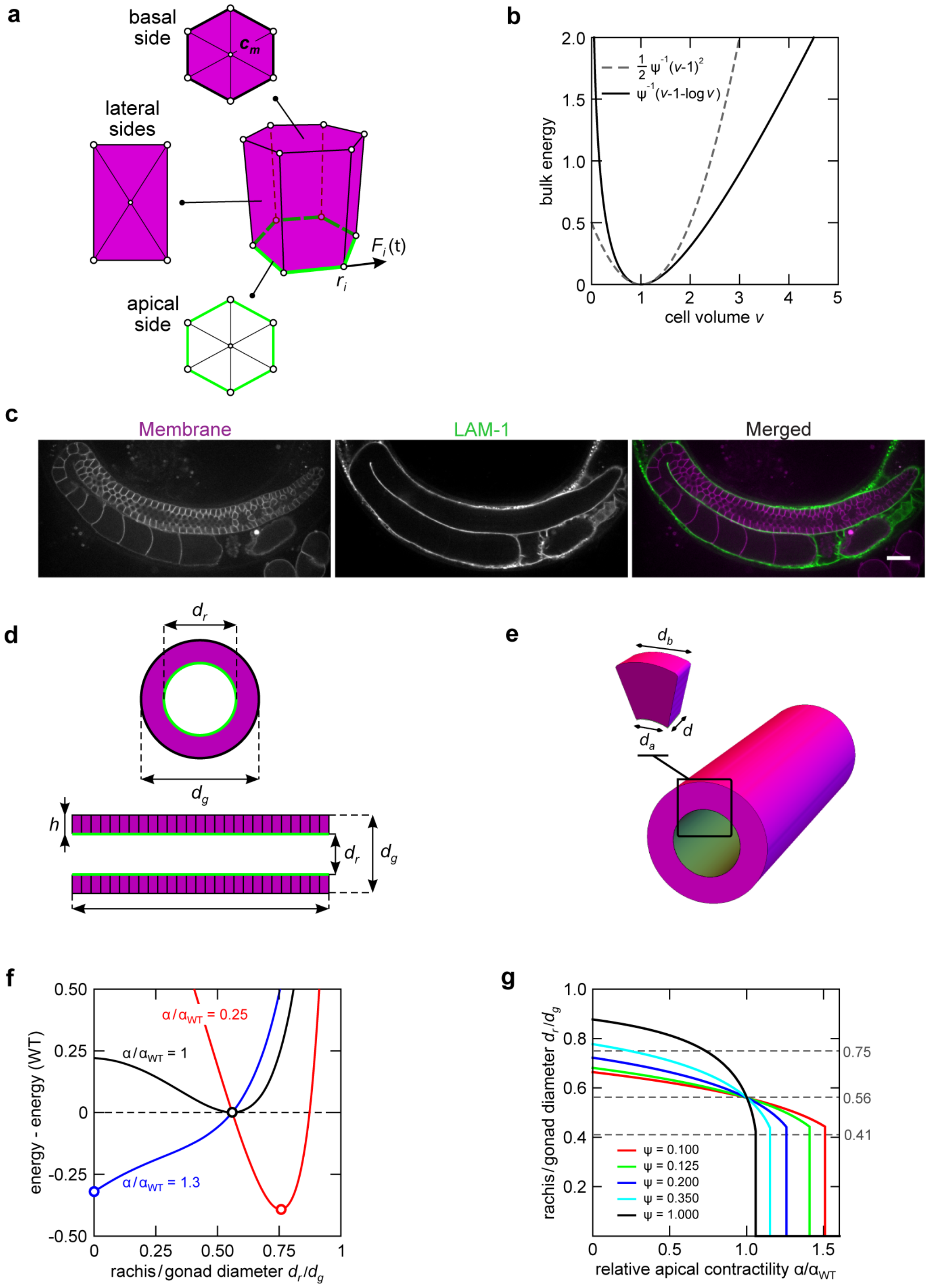

Supplementary Figure 4: Theoretical model of a smooth rachis tube. **(a)** Schematic of the 3D vertex model. Cells are parameterized by vertices $\boldsymbol{r}_i$ and can change shape due to forces on vertices $\boldsymbol{F}_i$. **(b)** Bulk-energy term $(1/2)\psi^{-1}(v-1-\log v)$ as a function of cell volume v [last term in Supplementary equation (3)]. The energy is minimal at the preferred cell volume ($v = 1$) and increases both if cell volume is increased or decreased. For small volumetric deformations from the preferred volume, the energy can be approximated by a parabola (dashed line). **(c)** Confocal image of the gonad expressing the laminin LAM-1::GFP (green) and membrane marker (magenta). Scale bar indicates 20 μm. **(d)** Geometry of the tube. The inner (rachis) and the outer (gonad) diameter is denoted by d_r and d_g, respectively, whereas the length of the tube is denoted by λ. **(e)** Schematic of a smooth cylinder used in the geometric approach [Supplementary equation (18)]. The apical and the basal dimensions of cells are denoted by d_a and d_b, respectively, whereas the transverse dimension is denoted by d. **(f)** Energy as a function of the relative rachis/gonad diameter d_r/d_g [Supplementary equation (18)]. At small apical contractility α the equilibrium apical diameter is finite, whereas at large α, the tube's apical diameter becomes essentially 0. **(g)** Relative rachis/gonad diameter d_r/d_g as a function of the relative apical contractility $\alpha/\alpha_{\mathrm{WT}}$. The apical diameter gradually decreases with an increasing α and drops to a small value at a critical α.

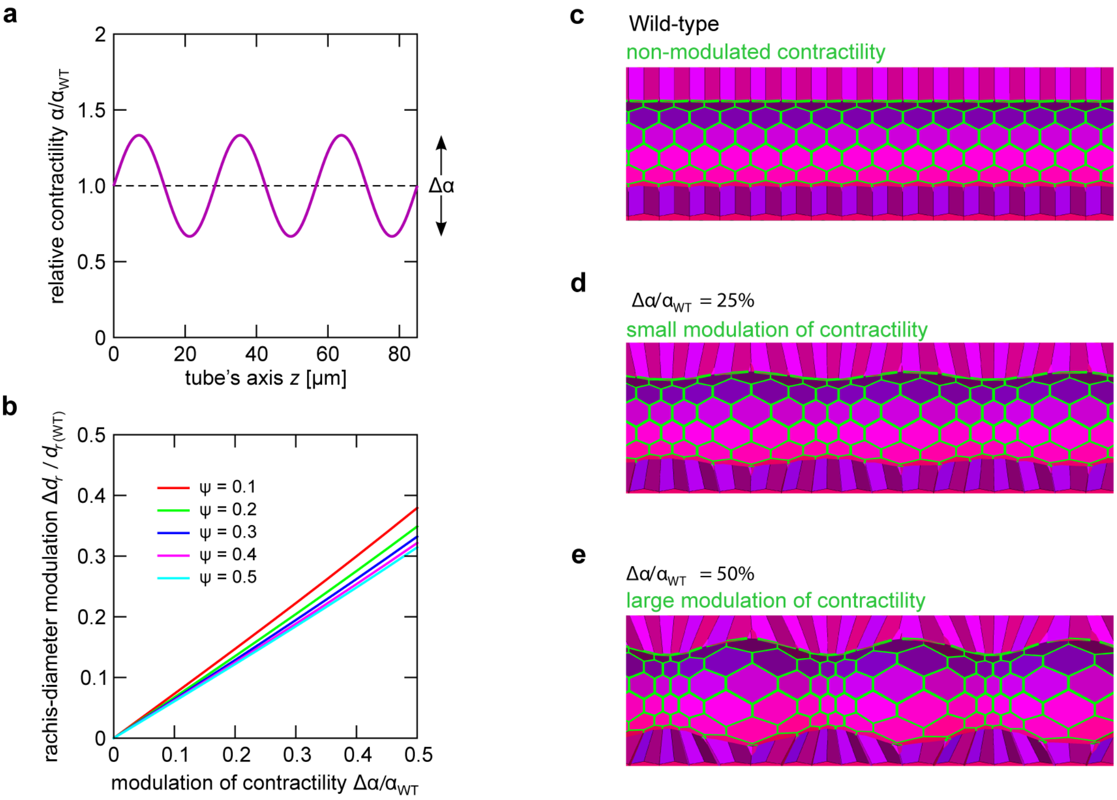

Supplementary Figure 5: Modulated germline shape with non-uniform tension. **(a)** Profile of the apical line tension α along the tub's axis z. **(b)** Modulation of the rachis diameter as a function of the modulation of the apical contractility at different values of cell compressibility. **(c-e)** Equilibrium configurations of the tube at different magnitudes of the contractility modulation at fixed cell compressibility ($\psi = 0.38$).

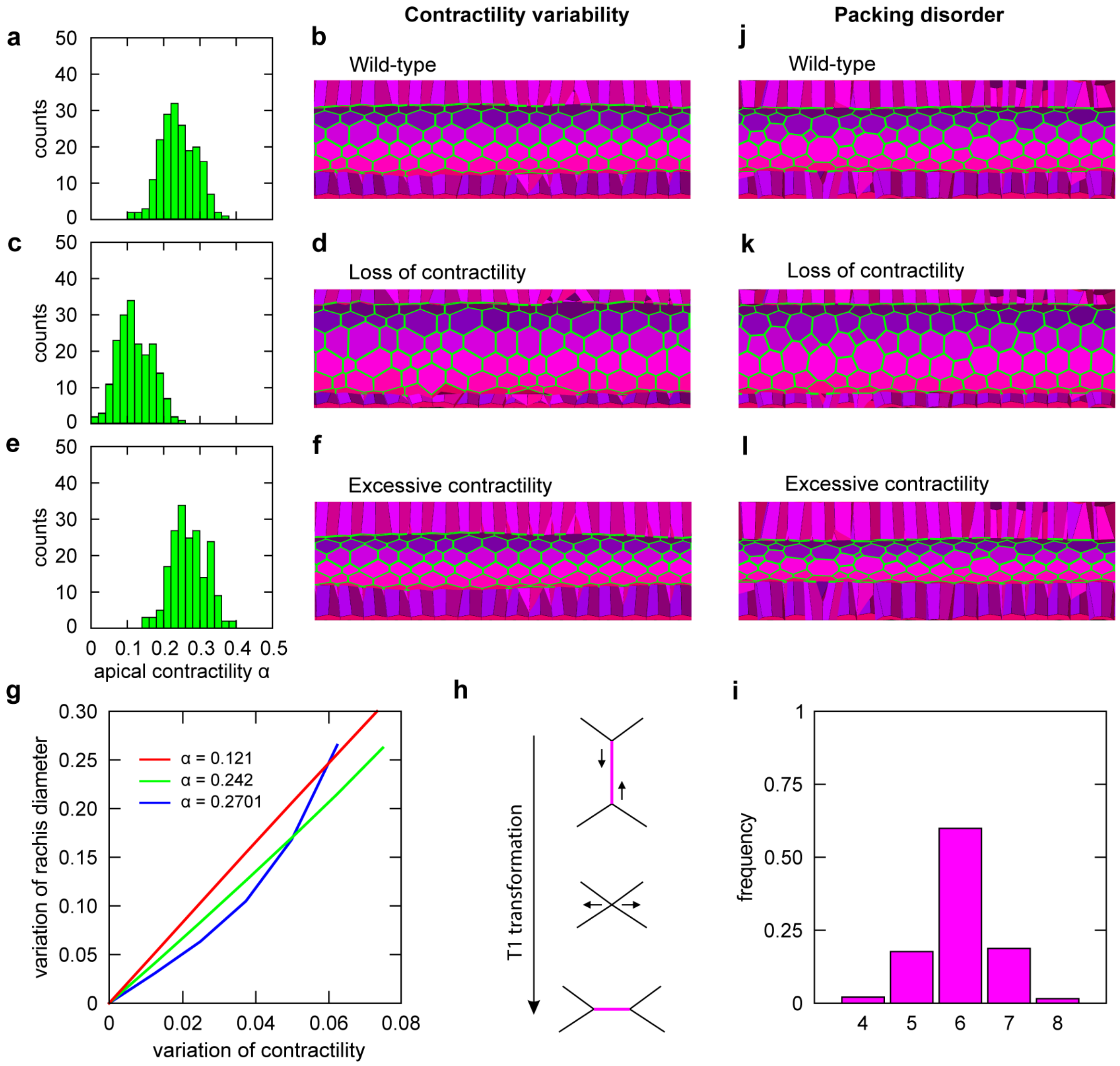

Supplementary Figure 6: Exploring the effect of irregularity of cell shape. Distributions of apical contractility shown in panels **(a)**, **(c)** and **(e)** give equilibrium shapes shown in panels **(b)**, **(d)**, and **(f)**, respectively. **(g)** Variation of rachis diameter as a function of the imposed variation of contractility. **(a)** Schematic of a T1 transformation. **(i)** The imposed distribution of polygons in tissues shown in panels **(j-l)**. **(j-l)** Equilibrium tissue shapes at $\psi = 0.38$, and $\alpha = 0.242$ **(j)**, $\alpha = 0.121$ **(k)** and $\alpha = 0.2701$ **(l)** and the distribution of polygons shown in panel **(i)**.

Supplementary Tables

Supplementary Table 1: The C. elegans strains used in this study

Strain	Genotype	Reference
UM208	*unc-119(ed3) III; ltIs81 [Ppie-1::gfp-TEV-Stag::ani-2; unc-119 (+)]; ltIs44 [Ppie-1::mCherry::PH(PLC1delta1); unc-119(+)] IV*	1
BU70	*lifeact ::RFP (zbIs2(Ppie-1lifeACT::RFP)*	Gift from Zhirong Bao's Lab
RZB 213	*plst-1(msn190[plst-1::GFP]) IV*	2
RZB88	*plst-1(tm4255) IV; ltIs37 [pAA64; pie-1::mCherry::his-58 + unc-119(+)] IV; ltIs38 [pAA1; pie-1::GFP::PH(PLC1delta1) + unc-119(+)]*	2
LP162	*nmy-2(cp13[nmy-2::GFP + LoxP]) I*	3
LP229	*nmy-2(cp52 [nmy-2::kate + LoxP unc-119 (+) Lox P]) I; unc-119 (ed3) III*	4
OD38	*unc-119(ed3) III; ltIs28[pASM14; pie-1/GFP-TEV-Stag::ANI-1; unc119(+)]*	5
COP1481	*unc-119(ed3) III; unc-59 (knu463 [unc-59::degron::mKate2 + loxP unc-119 (+) loxP]) I*	This study
COP1234	*cyk-4 (knu286 [cyk-4::GFP + loxP]) III*	This study
RZB282	*lifeact ::RFP (zbIs2(Ppie-1lifeACT::RFP); nmy-2(cp13[nmy-2::GFP + LoxP]) I*	This study
COP937	*unc-119(ed3) III; cyk-1 (knu84 [cyk-1::GFP + loxP unc-119 (+) loxP]) III*	This study
JCC389	*cyk-1(or596) III*	Gift from Julie Canman's Lab
RZB268	*cyk-1(or596ts)III; unc-119(ed3) III; ltIs81 [Ppie-1::gfp-TEV-Stag::ani-2; unc-119 (+)]; ltIs44 [Ppie-1::mCherry::PH(PLC1delta1); unc-119(+)] IV*	This study
UN1608	*nmy-1(xb5[nmy-1::mKate2]) X*	Gift from Erin Cram's Lab
RZB267	*plst-1(tm4255) IV;nmy-2 (cp52[nmy-2::mKate2]) I*	This study
RZB270	*plst-1(tm4255) IV; unc-119(ed3) III; ltIs81 [Ppie-1::gfp-TEV-Stag::ani-2; unc-119 (+)]; ltIs44 [Ppie-1::mCherry::PH(PLC1delta1); unc-119(+)] IV*	This study
OD95	*tIs37 [pAA64; pie-1::mCherry::his-58 + unc-119(+)] IV. ltIs38 [pAA1; pie-1::GFP::PH(PLC1delta1) + unc-119(+)]*	6
RZB266	*nmy-2 (cp52[nmy-2::mkate]) I;tIs38 [pAA1; pie-1::GFP::PH(PLC1delta1) + unc-119(+)].*	This study
NK651	*qyIs108 [lam-1p::lam-1::dendra + unc-119(+)] IV*	7
RZB319	*ltIs44 [Ppie-1::mCherry::PH(PLC1delta1); unc-119(+)]; unc-119(ed4) III; qyIs108 [lam-1p::lam-1::dendra + unc-119(+)] IV*	This study

Supplementary Table 2: Primers used for cloning *nmy-1* and *gck-1* into L4440 vector.

Gene	Primer	Primer sequence
nmy-1	Forward primer (5'-3') for *nmy-1*	CGACTCACTATAGGGAGACCGGCAGATC TGAATTGAATGGGATTTCATTGATTTCGG
	Reverse primer (5'-3') for *nmy-1*	CCCCTCGAGGTCGACGGTATCGATAAGC TTCTTTCCTCTTCAAGATGGGCAAG
	Forward primer (5'-3') for L4440 vector	GGAGTTCTTGCCCATCTTGAAGAGGAAA GAAGCTTATCGATACCGTCGACC
	Reverse primer (5'-3') for L4440 vector	GTCCGAAATCAATGAAATCCCATTCAATT CAGATCTGCCGGTCTCCCTATAG
gck-1	Forward primer (5'-3') for *gck-1*	CGACTCACTATAGGGAGACCGGCAGATC TGGTGCTACTACCATCACGCT
	Reverse primer (5'-3') for *gck-1*	CCCCTCGAGGTCGACGGTATCGATAAGC TTATCGAGGGCACCTTTCACAC
	Forward primer (5'-3') for L4440 vector	CCAATGCCTCGTGTGAAAGGTGCCCTCG ATAAGCTTATCGATACCGTCGACC
	Reverse primer (5'-3') for L4440 vector	TGGACTTCCAAGCGTGATGGTAGTAGCA CCAGATCTGCCGGTCTCCCTATAG

Supplementary Notes

Supplementary Note 1: 3D vertex model

Epithelial cell sheets consist of a single layer of tightly packed cells and can be modeled *in silico* using a three-dimensional (3D) vertex model. Here individual cells are parametrized by the positions of vertices $\boldsymbol{r}_i = (x_i, y_i, z_i)$ forming polyhedral bodies, which can change shape due to mechanical forces (Fig. 5a of the main text and Supplementary Figure 4a). Epithelial sheets are characterized by their packing structure, which is represented by a network of intercellular junctions.

Cell-shape changes are driven by mechanical forces exerted on vertices. The dynamics of vertices is typically subject to strong friction with the environment, e.g. the extracellular matrix, and the time evolution of the position of vertex i at $\boldsymbol{r}_i(t)$ is given by the overdamped equation of motion:

$$\eta_i \frac{\mathrm{d}\boldsymbol{r}_i(t)}{\mathrm{d}t} = \boldsymbol{F}_i(t) \,. \tag{1}$$

Here $\boldsymbol{F}_i(t)$ is the total force on vertex i at time t, and η_i denotes the friction drag coefficient of vertices moving with velocity $\mathrm{d}\boldsymbol{r}_i/\mathrm{d}t$ relative to the extracellular matrix; for $i = 1...\mathcal{V}$, where $\mathcal{V}$ is the number of vertices. The forces are assumed to drive the system towards the local minimum of the total potential energy W and can therefore be written as $\boldsymbol{F}_i = -\nabla_i W$, where $\nabla_i = (\partial/\partial x_i, \partial/\partial y_i, \partial/\partial z_i)$.

In our vertex model, different energy contributions are expressed in terms of the areas of the lateral cell sides A_l, the perimeter of the apical side P, and cell volume V as

$$W = \sum_{m=1}^{\mathcal{C}} \left[\frac{1}{2}\Gamma_l A_l + \Lambda_a P^2 + \Psi^{-1} \left(V - V_0 - V_0 \ln \frac{V}{V_0} \right) \right]_m \,, \tag{2}$$

where the sum goes over all $\mathcal{C}$ cells in the tissue. The first term describes the surface energy of the lateral cell sides. The corresponding effective surface tension Γ_l accounts for the contributions of the tension in the cellular cortex and the adhesion at cell-cell interfaces. For the sake of simplicity, each lateral surface is shared by two cells, hence the factor 1/2 in front of Γ_l. The second term describes the actomyosin ring at the apical sides of cells, where the model parameter Λ_a quantifies its contractility. Unlike in the model by Coffman et al. [8], which addresses radial shape changes of the rachis bridges, we here focus on the overall cell- and tissue-shape changes. Since in the vertex model, degrees of freedom are cell vertices, contractility of the rachis bridges can be most easily taken into account by assuming them completely open so that they coincide with apical cell-cell junctions. This simplification does not allow us to address questions related to the openings of rachis bridges, however it is not expected to have significant effect on 3D shape of cells and the global tissue shape. Finally, the last term describes cellular compressibility due to the various macromolecular constituents and intracellular fluids. In particular, among other components, cell interior contains the cellular cortex, network of microtubule, and the nucleus, which are all deformed when cell volume is changed. This can be described using the bulk-energy term of the liquid drop model [9], where cells are viewed as compressible bodies with a preferred volume V_0 and are characterized by the isothermal cell compressibility Ψ. When it comes to large volumetric deformations of cells, such a description is more accurate than the soft potential of the form $(V - V_0)^2$, typically used in similar models (Refs. [10, 11] and Supplementary Figure 4b). Note that the pressure difference between the interior and the exterior of cells can be calculated using Murnaghan equation of state $\Delta p = \psi^{-1} (V_0/V - 1)$ [9].

The main assumptions of our model pertain to the description of the environment constraining the epithelium at both sufraces of the tube as well as at the boundaries. In particular, we assume that at the basal side, cells adhere tightly to the basement membrane, which is much less deformable than the epithelium (Supplementary Figure 4g-i). Therefore, the basal surface (i.e. the outer surface) of the tissue is fixed and basal cells sides are assumed to have fixed surface areas, such that they do not contribute to

the force balance in the radial direction and are thus not included in Eq. (2). Furthermore, we assume that the analyzed segment of the tube does not change its legnth and that the number of constituent cells is constant, such that the size of the simulation box can be fixed and periodic boundary conditions can be assigned along the tube's axis. These assumptions render our model particularly simple, allowing us to capture the experimentally observed shape changes of the rachis tube using only a few model parameters as discussed bellow, and identify the relevant physical mechanisms that determine the overall structure of the system.

Supplementary Note 2: Details of implementation

The total potential energy [Eq. (2)] can be rewritten in dimensionless form by taking $V_0^{1/3}$ as the unit length and Γ_l as the unit surface tension such that

$$w(\boldsymbol{r}) = \sum_{m=1}^{\mathcal{C}} \left[\frac{1}{2}a_l + \alpha p^2 + \psi^{-1}\left(v - 1 - \ln v\right)\right]_m \,, \tag{3}$$

where $w = W/\left(\Gamma_l V_0^{2/3}\right)$, $p = P/V_0^{1/3}$, and $a_l = A_l/V_0^{2/3}$ are dimensionless energy, surface area of lateral sides, and perimeter of the apical ring, respectively, whereas the dimensionless model parameters

$$\alpha = \frac{\Lambda_a}{\Gamma_l} \,, \quad \text{and} \quad \psi = \frac{\Psi\,\Gamma_l}{V_0^{1/3}} \,. \tag{4}$$

Assuming that all vertices have the same friction drag coefficient, i.e. the environment relative to which the vertices are moving is homogeneous, $\eta_i = \eta$ for all i and the characteristic time scale $\tau = \eta/\Gamma_l$. By rescaling $t/\tau \rightarrow t$ and $\boldsymbol{r}_i/V_0^{1/3} \rightarrow \boldsymbol{r}_i$ we obtain the dimensionless discretized equation of motion which reads

$$\boldsymbol{r}_i(t + \Delta t) = \boldsymbol{r}_i(t) - \Delta t \nabla_i w(\boldsymbol{r}) \,. \tag{5}$$

Here we chose the explicit Forward-Euler scheme to discretize Eq. (1) and we used $\Delta t = 10^{-3}$.

In order to solve the equation of motion [Eq. (5)], we need to calculate forces on vertices (i.e. $-\nabla_i w$). This can be done by triangulating cell surfaces and introducing additional vertices at the centers of cell sides such that each triangular surface element consists of two real vertices and the center vertex. The position of center vertex m is given by

$$\boldsymbol{c}_m = \frac{1}{n_m} \sum_{i \in \text{cell side } m} \boldsymbol{r}_i \,, \tag{6}$$

where n_m is the number of edges of the cell side in question (Supplementary Figure 4a). A triangular surface element is defined as a trio of vertices oriented in a counterclockwise direction, e.g. $(\boldsymbol{r}_i, \boldsymbol{r}_j, \boldsymbol{c}_m)$. Note that the positions of center vertices need to be updated at each step of the simulation.

Total mechanical energy of the tissue is a function of different integral quantities related to cell shape. Say that the energy depends on an integral quantity q, which further depends on the position of vertex i at $\boldsymbol{r}_i$ $w = w\big(q(\boldsymbol{r}_i)\big)$. Then the contribution of the integral quantity q to the total force on vertex i is calculated as

$$\boldsymbol{F}_{qi} = -\nabla_i w\big(q(\boldsymbol{r}_i)\big) = -\frac{\partial w}{\partial q} \nabla_i q(\boldsymbol{r}_i) \,. \tag{7}$$

Within our model, integral quantities contributing to total forces on vertices are surface areas of the lateral cell sides a_l, perimeter of the apical side p, and cell volume v. The corresponding force contributions to vertex i of the lateral and apical sides and cell volume read $-(1/2)\nabla_i a_l$, $-2\alpha p \nabla_i p$, and $-\psi^{-1}\left(1 - 1/v\right)\nabla_i v$, respectively. Calculation of the gradients of integral quantities a_l, p, and v is nontrivial and is described below.

Surface area of a lateral cell side is calculated as a sum of surface areas of all triangular facets that constitute the lateral side:

$$a_l = \sum_{(i,j)\in\text{lateral side } m} A_{ijm} \ , \tag{8}$$

Here the surface area of a triangular surface element, defined by a trio of vertices $(\boldsymbol{r}_i, \boldsymbol{r}_j, \boldsymbol{c}_m)$, is calculated as $A_{ijm} = (1/2)\,|(\boldsymbol{r}_i - \boldsymbol{c}_m) \times (\boldsymbol{r}_j - \boldsymbol{c}_m)|$ and the corresponding gradient

$$\nabla_i A_{ijm} = \frac{(\boldsymbol{r}_j - \boldsymbol{c}_m) \times [(\boldsymbol{r}_i - \boldsymbol{c}_m) \times (\boldsymbol{r}_j - \boldsymbol{c}_m)]}{2\,|(\boldsymbol{r}_i - \boldsymbol{c}_m) \times (\boldsymbol{r}_j - \boldsymbol{c}_m)|} \ . \tag{9}$$

The perimeter of the apical side of cell m is calculated as a sum of apical edge lengths as

$$p = \sum_{(i,j)\in\text{apical side } m} L_{ij} \ , \tag{10}$$

where $L_{ij} = \sqrt{(\boldsymbol{r}_i - \boldsymbol{r}_j)\cdot(\boldsymbol{r}_i - \boldsymbol{r}_j)}$ is the length of the edge connecting vertices $\boldsymbol{r}_i$ and $\boldsymbol{r}_j$. The associated gradient reads

$$\nabla_i L_{ij} = \frac{\boldsymbol{r}_i - \boldsymbol{r}_j}{\sqrt{(\boldsymbol{r}_i - \boldsymbol{r}_j)\cdot(\boldsymbol{r}_i - \boldsymbol{r}_j)}} \ . \tag{11}$$

The total volume of a cell is calculated as sum over all facets defining the cell:

$$v = \sum_{(i,j,m)\in\text{cell}} \sigma_{ijm} V_{ijm} \ ,$$

where $\sigma_{ijm} = -1$ if the vertices of the surface element $(\boldsymbol{r}_i, \boldsymbol{r}_j, \boldsymbol{c}_m)$ are oriented such that the normal vector to the surface points inwards and $\sigma_{ijm} = 1$ otherwise. Volume of a tetrahedron defined by the surface element and the origin $(0,0,0)$, $V_{ijm} = (1/6)\,\boldsymbol{c}_m\cdot(\boldsymbol{r}_i \times \boldsymbol{r}_j)$. The gradient of the volume reads

$$\nabla_i V_{ijm} = \frac{1}{6}\boldsymbol{r}_j \times \boldsymbol{c}_m \ . \tag{12}$$

The basal side of the gonadal tube is fixed and thus the basal vertices need to obey the constraint $x_i^2 + y_i^2 = r_b^2$ for $i = 1, 2, \ldots \mathcal{V}$, where r_b is the basal radius of the tube. Following Ref. [12], we derive the displacement vector $\delta\boldsymbol{r}_i$ for vertex i, which at each time step cancels the difference of the vertex' position from the constraint $f = x_i^2 + y_i^2 - r_b^2$:

$$\delta\boldsymbol{r}_i = c_i \nabla_i f = 2c_i\,(x, y, 0) \ , \tag{13}$$

such that

$$\delta\boldsymbol{r}_i \cdot \nabla_i f = -f(\boldsymbol{r}_i) \ . \tag{14}$$

Combining both equations gives the unknown coefficient c_i:

$$c_i = -\frac{f(\boldsymbol{r}_i)}{\nabla_i f \cdot \nabla_i f} = \frac{1}{4}\left(\frac{r_b^2}{x_i^2 + y_i^2} - 1\right) \tag{15}$$

and displacements of vertices due to this constraint are calculated using Eq. (13).

Supplementary Note 3: Geometry of the tube

We model an $\approx 90\,\mu$m-long segment of the gonadal tube containing $N_x = 16$ cells along the tube's axis and $N_\varphi = 12$ cells along the circumference. The initial condition of the simulation corresponds to the wild-type tube, in which the ratio of the gonad diameter and the average cell height $d_g/h = 4.55$, whereas

the ratio of the tube's length and the average cell height $\lambda/h = 15.3$ (Supplementary Figure 4c). Cell volumes are normalized such that the total volume of the epithelium

$$V_{\mathrm{tot}} = N_x N_\varphi = \pi \left(d_g - h\right) h\lambda \,. \tag{16}$$

It immediately follows $h = 1.06$, $d_g = 4.82$, and $\lambda = 15.3$ (measured in the units of $V_0^{1/3}$). The rachis diameter of the tube is calculated as $d_r = d_g - 2h = 2.7$ (corresponding to ≈ 15 μm in real units). To further simplify the model, we assume that cells are packed in a regular hexagonal lattice. The results do not change much if the cellular packing is disordered (see Sec. 4). Equilibrium structures of the tube are computed at different values of model parameters α and ψ, where the energy [Eq. (3)] is minimized by following the vertex dynamics given by Eq. (5).

The phase diagram of minimal-energy states is plotted in $(\alpha/\alpha_{\mathrm{WT}}, \psi)$ plane, where $\alpha_{\mathrm{WT}} \approx 0.242$ is the apical contractility of the wild type, determined by matching the ratio of the rachis diameter and the gonad diameter with the average value $d_r/d_g = 0.56$, as observed in experiments (Fig. 5b and g of the main text). We find that the phase diagram contains two regimes: The wide-tube regime and the narrow-tube regime. In particular, the rachis diameter of the tube is mostly governed by the apical contractility α: With increasing α, the rachis diameter first gradually decreases until it continuously however abruptly drops to a much smaller value at a critical α (which depends weakly on ψ; Fig. 5g of the main text). The wild-type shape is found in the wide-tube regime and is denoted in the phase diagram by an isoline at $\alpha/\alpha_{\mathrm{WT}} = 1$, where $d_r/d_g = 0.56$ (Fig. 5g of the main text), corresponding to the experimental findings (Fig. 2g of the main text). Increasing and decreasing contractility at a fixed cell compressibility gives tubular morphologies with decreased and increased rachis diameter, respectively (see isolines with $r_a/r_g = 0.41$ and 0.75 in Fig. 5g of the main text).

Overall, the equilibrium morphology results from a competition of different force contributions. In particular, the energy term αp^2 describing the apical contractility tends towards decreasing the apical side of the tube, which due to geometric constraints can be achieved only if the rachis diameter is decreased. Since the basal diameter is fixed, a decrease of the rachis diameter leads to an increase of the cell volume, costing the bulk energy $\psi^{-1}(v - 1 - \ln v)$. In turn, if the apical contractility is decreased, the surface tension prevails over the apical contractility causing the lateral sides to shorten. This tug-of-war between surface tension, apical contractility, and incompressibility of cells aptly explains experimentally observed morphologies, which all reside in the narrow-tube regime of our model phase diagram (Fig. 5g of the main text). The abrupt transition from a wide-tube to a narrow-tube regime was not observed experimentally and thus might be either an artefact of the model or a region of parameters not accessible experimentally. To learn more about this, we develop a coarse-grained geometric model (see below).

Another mechanical aspect that plays an important role in our model are the external constraints. In particular, the overall integrity of the tube is maintained by cells being attached to a rigid basement membrane at the basal side. We tested this by releasing the tube from the fixed-basal-side constraint. In this case, the tube looses its integrity and collapses due to the imposed surface tensions and the contractility of the apical belt. Furthermore, due to the imposed contractility at the apical side, the tube exerts a compressive force at the boundaries of the simulation box, which would cause the tube to be compressed also in longitudinal direction if the analyzed portion of the tube would suddenly loose contact from the rest of the embryo.

In conclusion, using a 3D vertex model, we showed that many of the observed morphologies including those of the wild-type and mutant embryos can be explained using a relatively simple physical model, where only the most dominant forces are considered and described by only two dimensionless parameters. Furthermore, our results show that the overall structure and morphology of the rachis tube are governed by an interplay between intra-/inter-cellular forces as well as by the forces imposed by external constraints.

To better understand the results of the vertex model, and in particular the abrupt transition between

the two branches of solutions in the phase diagram, i.e. the narrow- and the wide-tube regime (Fig. 5b of the main text), we develop an analytical approach, in which the tube is approximated by a smooth single-cell-thick epithelial cylinder. To simplify the description, we assume that the tube consists of identical cells that are on average curved along the tube's circumference and flat along the tube's axis. Let the dimension of the cell base in the direction of zero curvature, i.e. cell depth, be d and the dimensions of the apex and the base along the circumferential direction of the tube be d_a and d_b, respectively (Supplementary Figure 4d). Then the surface area of the lateral sides $a_l \sim (d_a + d_b)h + 2dh\sqrt{1 + (d_a - d_b)^2/4h^2}$, the perimeter of the apical side $p_a \sim 2(d_a + d)$, and cell volume $v \sim (d_a + d_b)dh/2$. We further assume that just like in the vertex model, the number of cells along the tube's axis N_x and along the tube's polar angle N_φ are conserved, since there are no cell rearrangements. This gives the following geometric constraints:

$$d_b = \frac{\pi d_g}{N_\varphi}\ , \tag{17a}$$

$$d_a = \frac{\pi(d_g - 2h)}{N_\varphi}\ , \tag{17b}$$

$$d = \frac{\lambda}{N_x}\ . \tag{17c}$$

Taking into account the geometry and constraints, we can express the mechanical energy per cell with geometric parameters N_x, N_φ, d_g, λ and with the rachis diameter $d_r = d_g - 2h$ as

$$\begin{aligned} w =& 2\alpha\left[\frac{\pi d_r}{N_\varphi} + \frac{\lambda}{N_x}\right]^2 + \frac{\pi}{4N_\varphi}\left(d_g^2 - d_r^2\right) + \frac{\lambda(d_g - d_r)}{2N_x}\sqrt{1 + \left(\frac{\pi}{N_\varphi}\right)^2} + \\ &+\psi^{-1}\left\{\frac{\pi(d_g^2 - d_r^2)\lambda}{4N_xN_\varphi} - 1 - \ln\left[\frac{\pi(d_g^2 - d_r^2)\lambda}{4N_xN_\varphi}\right]\right\}\ . \end{aligned} \tag{18}$$

The equilibrium shape of the tube is obtained by minimizing w with respect to d_r at different values of model parameters α and ψ. This is done numerically using `Wolfram Mathematica`. We find that the global energy minimum can be found either at a finite d_r corresponding to the local minimum, or at $d_r = 0$ (the domain boundary)–depending on the model parameters: If α is too small the bulk energy term prevails and the equilibrium rachis diameter is finite (red curve in Supplementary Figure 4e); in turn, at large α, the global minimum switches to $d_r = 0$ (blue curve in Supplementary Figure 4e), corresponding to a very small (yet finite) d_r found in the vertex model. The dependance of the equilibrium rachis diameter is somewhat similar to that obtained by the vertex model (Supplementary Figure 4f and Fig. 5b of the main text), which is of course expected. Minor quantitative differences between them arise due to simplifications and assumptions behind both approaches (as discussed above).

Supplementary Note 4: Modulated morphologies

Surprisingly, by simply altering apico-basal tension differences, we do not find structures with a modulated apical surface, as might be expected due to an imposed apico-basal asymmetry [13]. This further confirms that the constraints, i.e. the rigidity of the tube's basal surface and a non-variable length of the tube, play an important role.

We tested whether a spatial variation of the apical contractility can induce such a deformation. For simplicity, we imposed a sine-like profile of α along the tube's axis (z): $\alpha(z) = \alpha_{\mathrm{WT}} + (\Delta\alpha/2)\sin(2\pi kz/\lambda)$, where k is the number of peaks and $\Delta\alpha$ is the magnitude of modulation (Supplementary Figure 5a). We varied $\Delta\alpha/\alpha_{\mathrm{WT}}$ between 0 and 0.5 and measured the modulation of the rachis diameter at four different values of cell compressibility ψ (Supplementary Figure 5b). The corresponding equilibrium structures are shown in Supplementary Figure 5c-e. Naturally, these shapes are perfectly regular due to an imposed regular, i.e. sine-like profile of contractility.

The irregularity of shapes seen in experiments can arise due to random variation of contractility, which can vary both along the tube's axis as well as along the circumference. To test this, we imposed randomly variable contractility, such that for each cell j

$$\alpha_j = \alpha_0 + \zeta_j(\sigma) , \tag{19}$$

where α_0 is the mean value of contractility (same for all cells) and $\zeta_j(\sigma)$ is a random variable drawn from the Normal distribution with zero mean and standard deviation σ. We computed equilibrium shapes at $\psi = 0.38$ and $\alpha_0 = 0.242$, 0.121, and 0.2701, corresponding to the wild type, reduced-contractility case, and excessive-contractility case, respectively, and we imposed $\sigma = 0.05$ (Supplementary Figure 6a, c, and e). We find that the final shapes of the rachis tube become somewhat less regular and the average rachis diameter can be slightly affected, however on average, we find that rachis shape is quite robust to this perturbation (Supplementary Figure 6b, d, and f). The variability of the apical contractility is reflected in the variability of the rachis diameter as shown in Supplementary Figure 6g.

Finally, shape irregularity can as well arise from a disordered cellular organization within the tissue. We performed simulations, in which not all cells were hexagons. The initial configuration was prepared by starting with a regular hexagonal packing and then performing 25 T1 transformations on randomly chosen cell-cell junctions (Ref. [14] and Supplementary Figure 6h). The resulting structure has a distribution of polygons peaked at hexagons, however also containing quadrilaterals, pentagons, heptagons, and octagons (Supplementary Figure 6i). We repeated the same set of simulations as before, i.e. at $\psi = 0.38$ and $\alpha = 0.242$, 0.121, and 0.2701, corresponding to the wild-type tube, reduced-contractility case, and excessive-contractility case, respectively. Results are somewhat similar as in the case of variable contractility: The overall shape remains unchanged when compared to the perfectly-ordered tube, but at the level of individual cells, we observe slight variability of cell heights and thus irregularity of the tube's shape (Supplementary Figure 6j-l).

These results show, that although details related to imperfections in terms of cell packing and cell-to-cell variability of mechanical properties might play a role when it comes to the detailed structure at the level of individual cells, overall morphology is quite robust to these features. This also justifies simplifications of the original model described in the main text, where we assumed that these details should not play an important role.

Supplementary references

[1] Amini, R. *et al.* C. elegans Anillin proteins regulate intercellular bridge stability and germline syncytial organization. *J Cell Biol* **209**, 467 (2015).

[2] Ding, W.Y. *et al.* Plastin increases cortical connectivity to facilitate robust polarization and timely cytokinesis. *J Cell Biol* **216**, 1371-1386 (2017).

[3] Dickinson, D.J., Ward, J.D., Reiner, D.J. & Goldstein, B. Streamlined Genome Engineering with a Self-Excising Drug Selection Cassette. *Genetics* **200**, 1035-1049 (2015).

[4] Heppert, J.K. *et al.* Comparative assessment of fluorescent proteins for in vivo imaging in an animal model system. *Mol Biol Cell* **27**, 3385-3394 (2016).

[5] Maddox, A.S., Lewellyn, L., Desai, A. & Oegema, K. Anillin and the septins promote asymmetric ingression of the cytokinetic furrow. *Dev Cell* **12**, 827-835 (2007).

[6] McNally, K., Audhya, A., Oegema, K. & McNally, F.J. Katanin controls mitotic and meiotic spindle length. *J Cell Biol* **175**, 881-891 (2006).

[7] Ihara, S. *et al.* Basement membrane sliding and targeted adhesion remodels tissue boundaries during uterine-vulval attachment in Caenorhabditis elegans. *Nat Cell Biol* **13**, 641-651 (2011).

[8] Coffman, V.C., Kachur, T.M., Pilgrim, D.B, & Dawes, A.T. Antagonistic Behaviors of NMY-1 and NMY-2 Maintain Ring Channels in the C. elegans Gonad. *Biophys J* **111**, 2202-2213 (2016).

[9] Riest, J., Athanasopoulou, L., Egorov, S. A., Likos, C. N., & Ziherl, P. Elasticity of polymeric nanocolloidal particles. *Sci Rep* **5**, 15854 (2015).

[10] Misra, M., Audoly, B., Kevrekidis, I.G. & Shvartsman, S.Y. Shape Transformations of Epithelial Shells. *Biophys J* **110**, 1670-1678 (2016).

[11] Bielmeier, C. et al. Interface Contractility between Differently Fated Cells Drives Cell Elimination and Cyst Formation. *Curr Biol* **26**, 563-574 (2016).

[12] Brakke, K. The Surface Evolver. *Exp. Math.* **1**, 145-165 (1992); the Surface Evolver is available at http://www.susqu.edu/facstaff /brakke/evolver/evolver.html.

[13] Krajnc, M., & Ziherl, P. Theory of epithelial elasticity. *Phys Rev E* **92**, 052713 (2015).

[14] Krajnc, M., Dasgupta, S., Ziherl, P. & Prost, J. Fluidization of epithelial sheets by active cell rearrangements. *Phys Rev E* **98**, 022409 (2018).
