# Supplementary material for: Syncytial germline architecture is actively maintained by contraction of an internal actomyosin corset
Source: Nat Commun. 2018 Nov 8;9:4694. doi: 10.1038/s41467-018-07149-2 (PMC6224597; doi:10.1038/s41467-018-07149-2)
Supplement: Supplementary file 3 — Description of Additional Supplementary Files [file 41467_2018_7149_MOESM3_ESM.docx]

**Description of Additional Supplementary Files**

**File Name:** Supplementary Movie 1

**Description:** 3D reconstruction of a wild-type gonad performed using Imaris8.4 software. This is a representation of the 3D structure of a gonad expressing GFP::ANI-2 (green) and mCherry::PH (magenta) . Surface creation was done for a small region of interest as shown. Left end of the image is the proximal end of the gonad while right end represents distal region. Scale bar represents 10µm.

**File Name:** Supplementary Movie 2

**Description:** FRAP experiment on GFP::ANI-2, NMY-2::mKate and PLST-1::GFP. Midplane view of the representative FRAP images of GFP::ANI-2, NMY-2::mKate and PLST1::GFP. Photobleaching was carried out at the rachis bridge (rectangular box). Images were acquired at the rate of 30 seconds interval after photobleaching for 15 frames. Scale bar represents 5µm. The movie is displayed at a rate of 5 frames per second.

**File Name:** Supplementary Movie 3

**Description:** Laser incision at the rachis of control and nmy-2(RNAi) worms co-expressing GFP::ANI-2 and mCherry::PH. Line ablation (~ 9µm) was performed along the distal to proximal axis. Images were acquired at the rate of 1 frame per second. Scale bar represents 5µm. The movie is displayed at a rate of 5 frames per second.

**File Name:** Supplementary Movie 4

**Description:** Point ablation at the rachis bridge of the distal and proximal end of the gonads expressing GFP::ANI-2 and mCherry::PH. Images were acquired at the rate of 1 frame per second. Scale bar represents 5µm. The movie is displayed at a rate of 5 frames per second.

**File Name:** Supplementary Movie 5

**Description:** Point ablation at the rachis surface in the distal region of the gonad (without sectioning rachis bridge) and line ablation at the rachis surface perpendicular to the distal- proximal axis of the gonad expressing GFP::ANI-2 and mCherry::PH. Images were acquired at the rate of 1 frame per second. Scale bar represents 5µm. The movie is displayed at a rate of 5 frames per second.

**File Name:** Supplementary Movie 6

**Description:** Line ablation at the germ cell membranes along the distal-proximal axis of the gonad expressing GFP::ANI-2 and mCherry::PH. Images were acquired at the rate of 1 frame per second. Scale bar represents 5µm. The movie is displayed at a rate of 10 frames per second.

**File Name:** Supplementary Movie 7

**Description:** Cytoplasmic streaming in the distal region of the gonads of control, cyk1(or596) mutant and nmy-2(RNAi) on L4 stage worms. Differential interference contrast (DIC) images were acquired at the rate of 2 seconds per frame focusing on the mid-plane view showing several cytoplasmic particles. All the images are aligned in the direction of distal (left) to the proximal axis (right). Scale bar represents 5µm. The movie is displayed at a rate of 10 frames per second.

**File Name:** Supplementary Movie 8

**Description:** Cytoplasmic streaming in the distal region of the gonads of cyk-1(RNAi) on cyk1(or596) mutant, nmy-2(RNAi) on L3 stage worms and mel-11(RNAi) worms. Representative DIC images acquired at the rate of 2 seconds per frame of the indicated genotypes. All the images are aligned in the direction of distal (left) to the proximal axis (right). Scale bar represents 5µm. The movie is displayed at a rate of 10 frames per second.

**File Name:** Supplementary Movie 9

**Description:** Time evolution of the shape of gonadal tube at a reduced apical contractility compared to the wild-type α=0.121, computed using a 3D vertex model. The value of cell compressibility is ψ=0.38. The rachis diameter of the tube increases with time, while the cell height decreases correspondingly. Green hexagons represent germ cells’ apical ends.

**File Name:** Supplementary Movie 10

**Description:** Time evolution of the shape of gonadal tube at an increased apical contractility compared to the wild-type α=0.2701, computed using a 3D vertex model. The value of cell compressibility is ψ=0.38. The rachis diameter of the tube decreases with time, while the cell height increases correspondingly. Green hexagons represent germ cells’ apical ends.

**File Name:** Supplementary Movie 11

**Description:** Time evolution of the shape of gonadal tube corresponding to a sine-like modulation of tension along the tube’s axis computed using a 3D vertex model. The average value and the magnitude of modulation of the tension are set to α=0.242, and Δα=0.25, respectively. The value of cell compressibility is ψ=0.38. The apical surface of the tube responds to the applied modulation of tension by assuming a mildly pearled shape. Green hexagons represent germ cells’ apical ends.

**File Name:** Supplementary Movie 12

**Description:** Time evolution of the shape of gonadal tube corresponding to a sine-like modulation of tension along the tube’s axis computed using a 3D vertex model. The average value and the magnitude of modulation of the tension are set to α=0.242, and Δα=0.5, respectively. The value of cell compressibility is ψ=0.38. The apical surface of the tube responds to the applied modulation of tension by assuming a strongly pearled shape. Green hexagons represent germ cells’ apical ends.
